# Alternative Systems: The Interplay Between Criminal Groups’ Influence and Political Trust on Civic Honesty in the Global Context

**DOI:** 10.1177/19485506231176615

**Published:** 2023-06-07

**Authors:** Giovanni A. Travaglino, Pascal Burgmer, Alberto Mirisola

**Affiliations:** 1 Royal Holloway, University of London, Egham, UK; 2 University of Southampton, UK; 3 University of Palermo, Italy

**Keywords:** civic honesty, public goods, political trust, organized criminal groups

## Abstract

Individuals’ endorsement of standards of civic honesty is necessary for democracies to flourish. A critical driver of civic honesty is the relationship of trust between individuals and institutions. Research has yet to systematically assess the contextual factors that may moderate this relationship. In this study, we examined the societal influence of organized criminal groups. Criminal groups operate as alternative systems of authority that erode the reliability of institutions’ moral standards. We employed a new indicator that quantifies their societal influence to test the hypothesis that the association between individuals’ political trust and civic honesty would weaken in countries more strongly affected by criminal groups. Multilevel evidence across 83 representative national samples (*N* = 128,839) supported this hypothesis. Moreover, the association between political trust and civic honesty was negative in contexts where criminal groups’ influence was more extreme. We discuss the implications of the findings and future research directions.

Dishonesty in the context of public goods has significant negative consequences for societies (Ariely et al., 2019; Cohn et al., 2019; Yamagishi, 1986). For instance, tax avoidance costs governments worldwide over US$483 billion annually (Tax Justice Network, 2021), money that could be spent on health care, education, or tackling global challenges. Behaviors such as welfare fraud and corruption are highly detrimental to individuals and societies, diverting resources away from programs that could alleviate inequality and ultimately save lives (Azfar et al., 2001; Q. Li et al., 2018). Even relatively minor acts of fraud, such as fare evasion on public transport, can lead to considerable adverse outcomes, affecting the long-term sustainability of public services (Bucciol et al., 2013). Given their great relevance, the conditions leading individuals to endorse or reject moral standards of honesty in the context of common goods have been a topic of inquiry in a wide range of disciplines, including psychology (Tyler, 2006; Van Lange et al., 2013; Vauclair & Fischer, 2011), criminology (LaFree, 1998), economics (Besley, 2020; Knack & Keefer, 1997), and political sciences (Ariely et al., 2019; Putnam et al., 1994).^1^

Research indicates that a critical driver of civic honesty is the extent to which individuals trust political and legal authorities and institutions (LaFree, 1998; Letki, 2006; Levi et al., 2009; Nivette, 2014; Tyler, 2006). Political trust constitutes the foundation of the social contract between citizens and institutions. This social contract leads citizens to perform their civic duty in exchange for good administration (Besley, 2020; Levi & Stoker, 2000). However, prior research has also shown the existence of substantial heterogeneity across countries in the relationship between political trust and civic honesty (e.g., Chan et al., 2017; Travaglino & Moon, 2021). In this research, we examined the question of what might explain this heterogeneity by investigating a contextual factor that may profoundly alter the nature of the social contract between citizens and institutions, namely, the extent to which countries are influenced by organized criminal groups.

Criminal groups such as mafias and criminal organizations have a strong economic impact worldwide. Because they are “organized,” these groups also have substantial social and political influence, corroding the morality of institutions and establishing alternative systems of authority (e.g., Barnes, 2017; Ferreira & Gonçalves, 2022; Lessing, 2020; Travaglino & Abrams, 2019). In this research, we leveraged an indicator developed by the Global Initiative Against Transnational Organized Crime (GI-TOC; https://globalinitiative.net/), quantifying the degree of social, economic, and political influence of criminal groups across countries and territories. We used this indicator and representative samples from 83 nations included in the joint European Values Study and World Values Survey (EVS/WVS, 2022) to investigate how the relationship between individuals’ political trust and their moral standards of civic honesty may be moderated by cross-country differences in the influence of criminal groups.

## Civic Honesty and Political Trust

In the context of public goods, individuals face a dilemma between self and collective interests (Van Lange et al., 2013). Public goods are commodities and services from which individuals cannot be barred. Because individuals can use a public good without sharing the costs, the most rational individual response is free riding. For instance, individuals can benefit from government services while deciding to evade the taxes that make those services possible. Free riding affects the long-term viability of public goods, and societies attempt to limit this behavior by devising systems of punishment (i.e., legal sanctions and surveillance). Such systems are costly, inefficient, and sometimes ineffective (Balliet & Van Lange, 2013; X. Li et al., 2018; Zelditch, 2006).

Despite the rational appeal of free-riding, there is also evidence that individuals act honestly by contributing readily to public goods in the absence of sanctions (Fehr & Fischbacher, 2002). Individuals can internalize moral standards of civic honesty toward public goods, leading them to endorse attitudes that maximize the collective interest (Letki, 2006). Endorsing these standards implies that individuals are less likely to justify and ultimately engage in illegal behaviors (Kirchler et al., 2008; Knechel & Mintchik, 2022; Letki, 2006).

Trust in political authorities and institutions is especially important in shaping individuals’ moral standards of civic honesty. Political trust is a critical feature of the relationship between individuals and institutions (Levi & Stoker, 2000; Norris, 2022) and a crucial component of institutions’ legitimacy (Citrin & Stoker, 2018; Tyler, 2006). Research indicates that stronger political trust is associated with a reduced need for sanctions and surveillance because individuals are more likely to comply voluntarily with authorities’ requests (Jackson, 2013; Tyler, 2006; Tyler & Huo, 2002). For instance, recent cross-national research has shown that political trust is linked to individuals’ willingness to comply with health policies in the context of the COVID-19 pandemic (Devine et al., 2021; Lalot et al., 2022; Pagliaro et al., 2021; Travaglino & Moon, 2021).

Cross-national analyses of several countries and regions have shown that political trust positively predicts individuals’ endorsement of civic honesty (Chan et al., 2017; Letki, 2006; Marien & Hooghe, 2011). These studies revealed that political trust is a stronger predictor of civic honesty than “horizontal” forms of trust, namely, trust in fellow citizens. The latter finding is consistent with the idea that authorities play a crucial role in upholding the social contract, fostering positive relationships of reciprocity between citizens and the state (Besley, 2020).

Importantly, however, these studies also revealed the existence of cross-country heterogeneity in the relationship between political trust and civic honesty. For instance, using data from the 2010–2015 wave of the WVS/EVS, Chan et al. (2017) investigated the association between political trust and a specific instance of civic honesty, namely, the justifiability of tax evasion, across 108 countries. Although political trust positively predicted civic honesty in most countries, they also found weaker, null, and negative associations between the two constructs. Research has yet to systematically address the important question of what may explain this heterogeneity (Chan et al., 2017). In this study, we focussed on cross-country differences in the influence of criminal groups because of these groups’ distinctive capacity to alter the nature of the social contract between citizens and institutions.

## The Influence of Organized Criminal Groups

Organized crime is a concept notoriously difficult to define (von Lampe, 2016). In this article, we employed the GI-TOC’s definition of organized crime as illegal activities conducted nationally or transnationally by groups or networks to obtain a financial or material benefit (*Methodology*, n.d.). The advantage of using this definition is that it allows quantifying the social impact of various kinds of criminal actors. Accordingly, we employ the label “Criminal Groups” to refer to a broad range of groups, including *mafia-style groups* (structured hierarchical groups with a known name and identifiable membership, also including militias funded by illicit activities), *criminal networks* (smaller and loosely associated groups of criminals without known name or clear leadership structure), *state-embedded* (criminals embedded in the state) and *foreign actors* (criminals operating outside their home country). Criminal groups are involved in the illegal trafficking of drugs and weapons. They place a great burden on societies, increasing global economic costs and security risks.

Criminal groups are an especially critical factor because they directly erode political and legal institutions, reducing the quality of democracy in a country (Allum & Siebert, 2003; Pinotti, 2015; Sung, 2004). Unlike terroristic organizations, typically criminal groups do not have an explicit political agenda. However, in contrast to “disorganized” forms of crime, they can become deeply embedded within societies. To date, little quantitative research has systematically assessed the social implications of these groups’ influence (Pinotti, 2015; e.g., Van Dijk, 2007). This gap is especially conspicuous in psychology, where research has focussed almost exclusively on individuals’ perceptions of legal institutions while paying less attention to the implications of organized criminal groups for individuals’ moral and political attitudes (Travaglino & Abrams, 2019).

This gap may in part be due to the challenges associated with quantifying the influence of criminal groups across societies (Hall, 2018; Holmes, 2016). The clandestine nature of these groups and the existence of a large number of different legal definitions have complicated official efforts to measure the phenomenon (Hall, 2018). Consequently, there have been very few systematic attempts to create indicators of organized crime’s impact (e.g., Pinotti, 2015). The novel GI-TOC Index employed in this study is grounded in a shared and broad definition of criminal groups. Moreover, the index benefits from regional experts’ local assessments and harmonization of the scores across contexts (*Methodology*, n.d.). These features allow researchers to test hypotheses on how cross-national differences in criminal groups’ influence may be linked to individuals’ attitudes in crucial domains.

## The Present Study

Criminal groups destabilize the social contract between institutions and citizens by colluding with, influencing, and subverting the moral mandate of public bodies (Allum & Siebert, 2003; Van Dijk, 2007). They can establish alternative systems of authority capable of undermining governments’ prerogatives. For instance, they are able to replace the state in offering protection, affirming norms, or managing relationships and exchanges within communities (Gambetta, 1996; Lessing, 2020; Travaglino & Abrams, 2019).

Criminal groups can affect institutions both locally (Kirby et al., 2018) and nationally (Maruko, 2003) because they perform duties that should be fulfilled by the state. Their ability to influence institutions may lead to the view that the government has lost control over some functions or geographical areas. People need not be directly exposed to the threat of criminal groups, or become involved in illegal activities, to be aware of these groups’ influence on political and legal institutions (cf. Sobering & Auyero, 2022). This is because criminal groups’ actions are widely reported in the media (Di Ronco & Lavorgna, 2018). Therefore, in the present research, we tested the hypothesis that the increase in the influence of criminal groups across countries would be linked to a weaker association between political trust and civic honesty.

## Methods

### Participants

We tested our hypothesis using representative samples from the joint European Values Study and World Values Survey v.3.0 (EVS/WVS, 2022). We included in our analyses all the countries for which there were available data (Wave 7, survey period 2017–2022). The sample consisted of 128,839 participants (53.4% female, 46.6% male, *M*_age_ = 45.29, *SD*_age_ = 17.01) nested in 83 countries (eight countries from Africa, 13 from the Americas, 26 from Asia, 34 from Europe, and two from Oceania). No power analysis was conducted: the sample size depended on the data available.

### Measures

#### Individual-Level Variables

##### Endorsement of Civic Honesty

Four items in the WVS/EVS drawn from the Morally Debatable Behaviors Scale (Harding et al., 1986) measured individuals’ moral standards of honesty in the civic context (e.g., Letki, 2006; Vauclair & Fischer, 2011). Participants were asked the extent to which each of the following behaviors was justifiable, “Someone accepting a bribe in the course of their duties,”“Cheating on tax if you have the chance,”“Avoiding a fare on public transport,” and “Claiming state benefits which you are not entitled to” (1 = *never justifiable* to 10 = *always justifiable*). Items were reversed and averaged (α = .75). Higher scores indicated a stronger endorsement of civic honesty.

##### Trust in Political and Legal Authorities

To measure political trust, we used items tapping into individuals’ confidence in six domestic institutions (as in Newton & Zmerli, 2011), “Parliament,”“The Police,”“The Civil Service,”“The Government,”“The Political Parties,”“The Justice System/Courts” (1 = *a great deal* to 4 = *none at all*). Items were reversed and averaged (α = .88). Higher scores indicated stronger political trust.

##### Demographics

Guided by previous research (e.g., Letki, 2006; Marien & Hooghe, 2011), in the analyses, we controlled for several individual-level variables associated with the endorsement of civic honesty. We included gender (recoded as −1 = men, 1 = women) and measures of age, income, and education. The measure of income used the scales of the WVS (a respondent’s assessment of the household income ranging from 1 [*lowest income group*] to 10 [*highest*]) and the EVS (a respondent’s assessment of the decile to which the household income belongs). The measure of education indicated the highest level of education attained by respondents using the ISCED-code one digit (0 = *less than primary* to 8 = *doctoral or equivalent*).^2^

#### Country-Level Indicators

##### Influence of Criminal Groups

We used the “Criminal Actors” dimension of the *Global Organized Crime Index* (ocindex.net). This indicator quantifies criminal groups’ social, political, and economic impact across nations and territories. The index was built using extensive reviews of objective evidence, expert-led assessments of countries’ circumstances, regional-expert group meetings, and internal calibration of scores (see *Methodology*, n.d.). The indicator’s score ranges from 1 (*nonexistent to little influence*) to 10 (*severe influence*) and rates countries and territories on the impact of different types of criminal groups, ranging from well-defined and structured organizations (mafia-style groups, foreign mafias, and guerrilla and militia groups primarily funded by illicit activities) to more lose networks of organized criminals.

##### Additional Country-Level Indicators

We sought to control for other country-level differences that may predict individuals’ standards of civic honesty (Letki, 2006; Marien & Hooghe, 2011). We controlled for two indicators of the state of the economy, the Human Development Index (HDI) and the Gross Domestic Product per capita (GDPpc). The HDI is published by the United Nations Development Program (UNDP) and is a composite index of life expectancy at birth, average education level (mean years of schooling completed), and gross national income. The HDI data (range: 0–1) were retrieved from the UNDP’s website (https://hdr.undp.org/data-center). The GDPpc data (in US$) were retrieved from the World Bank Institute website (https://data.worldbank.org/indicator/NY.GDP.PCAP.CD). For both indicators, we used countries’ latest available year.

Because prior research has highlighted the importance of voice in individuals’ compliance with legal standards (Tyler & Jackson, 2014), we also included an indicator of societies’ levels of voice and accountability (range: −2.5 to 2.5). This indicator tapped countries’ freedom of expression, participation, and free media. Finally, because the influence of criminal groups may be linked to other sources of instability (Makarenko, 2004), we included an indicator of countries’ stability (range: -2.5 to 2.5). This indicator quantifies countries’ general political stability and level of terroristic threats. The indicators of voice and stability were developed by the World Bank Institute (Kaufmann et al., 1999).

## Results

### Preliminary Analyses

**Figure 1:**
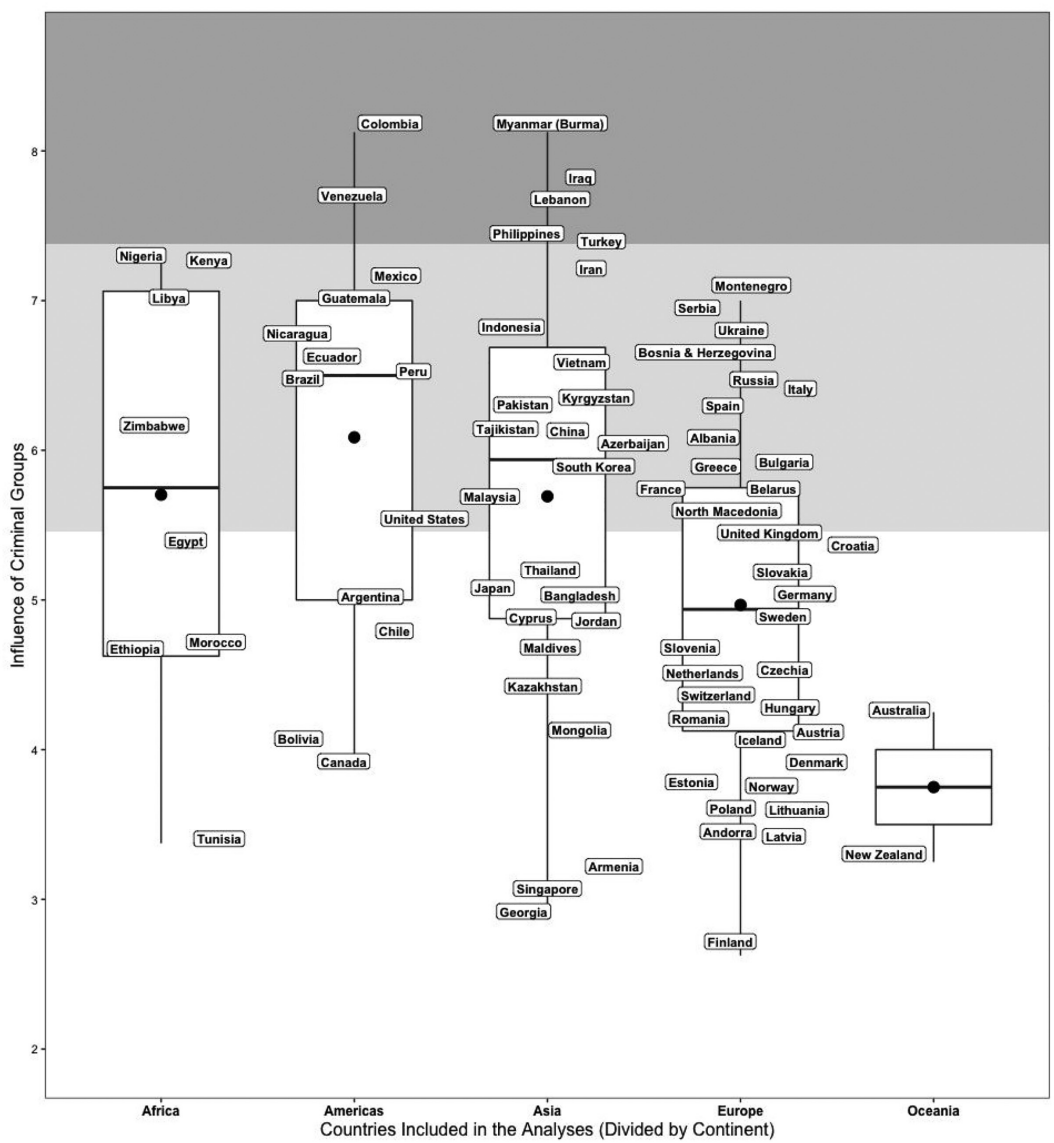
Country Distribution of the Influence of Criminal Groups Indicator *Note.* The dots at the center of the boxplots indicate the average degree of influence of criminal groups in each continent (the lines indicate the median). The three bands represent the Johnson–Neyman intervals for the cross-level interaction: in the white band, the predicted relationship between political trust and civic honesty was significant and positive; in the light gray band, this relationship was nonsignificant, and in the dark gray band, it was significant and negative (further details are provided in the text).

Figure 1 displays how all the countries included in the study scored on the influence of criminal groups indicator. To enhance clarity, the figure presents the countries divided by continent. In our samples, the country with the lowest score was Finland (2.63), while the countries with the highest score were Myanmar and Colombia (8.13). Table 1 summarizes mean values, standard deviations, and correlations among the variables. The overall correlation between political trust and civic honesty was small but significant (*r* = .05, *p* < .001). This correlation, however, does not consider cross-country heterogeneity in the association between the two constructs. We investigated this heterogeneity using a multilevel approach.

**Table 1:** Mean Values, Standard Deviations, and Correlations for Country-Level Indicators and Individual-Level Variables

Country-level indicators	*M*	*SD*	1	2	3	4	5
1. Criminal Groups	5.41	1.39	—				
2. HDI	0.80	0.11	−.51***	—			
3. GDPpc (US$)	20,983.81	22,967.33	−.50***	.78***	—		
4. Voice	0.12	1.01	−.55***	.79***	.74***	—	
5. Stability	−0.09	0.95	−.67***	.82***	.72***	.81***	—
Individual-level variables	*M*	*SD*	6	7	8	9	10
6. Civic Honesty	8.63	1.69	—				
7. Political Trust	2.35	0.71	.05***	—			
8. Education	3.68	1.97	.01***	−.01***	—		
9. Income	4.91	2.35	.007**	.04***	.30***	—	
10. Age	45.29	17.01	.17***	.04***	−.10***	−.12***	—
11. Gender	—	—	.03***	.01***	−.02***	−.06***	.0002

*Note*. *SD* = Standard Deviation; HDI = Human Development Index; GDPpc = Gross Domestic Product per capita (in US$). Gender was coded as −1 = male and 1 = female.

** *p* < .01. ***p < .001.

### Testing the Multilevel Model

To test our hypothesis that country-level differences in the influence of criminal groups moderated the association between individuals’ political trust and civic honesty, we used a multilevel model in which participants (Level 1) were nested within countries (Level 2). The intercept of civic honesty was allowed to vary across countries. Political trust was part of the cross-level interaction, and we included the random slope for this variable in the model (as recommended by Heisig & Schaeffer, 2019).

Level 1 variables were group-mean centered (Enders & Tofighi, 2007; Hox et al., 2017). Group centering removes the effects of between-country variation from the Level 1 variables, yielding pure within-country estimates of the associations. We reintroduced the between-country effects of political trust in the model by including countries’ aggregated scores for this variable. Doing so enabled us to examine (and control for) the associations between political trust and civic honesty at both hierarchical levels.

We built our model using the stepwise approach recommended by Hox et al. (2017). First, we estimated an empty model without explanatory variables. The empty model provided a benchmark for subsequent steps and was used to calculate the intraclass correlation (ICC). ICC indicates the proportion of total variance explained by the grouping structure in the population (Hox et al., 2017). In the current study, the ICC was .15 (a medium effect size), confirming the suitability of the multilevel approach. We then tested a model including all Level 1 (individual) variables, followed by a model in which we added all Level 2 (country) indicators. Next, we tested a model including the random slope of political trust, followed by a model which also added the cross-level interaction between the influence of criminal groups indicator and political trust. We used chi-squares to test fit improvements for the nested models. The results of these tests indicate significant improvement in model fit across steps (see Table 2).

**Table 2:** Model Fit Changes ( ${\text{χ}}^{\text{2}}$ of Δ Deviance)

Models	$\chi^{2}$ (*df*)	AIC	BIC
Model 0	−	481,249	481,278
Model 1	2,769.72 (5)***	478,489	478,567
Model 2	19.53 (6)**	478,482	478,619
Model 3	792.66 (2)***	477,693	477,849
Model 4	16.06 (1)***	477,679	477,845

*Note*. Model 0 was the intercept-only model; Model 1 added the Level 1 variables; Model 2 added the Level 2 indicators; Model 3 added the random slope of Political Trust; Model 4 added the cross-level interaction. $\chi^{2}$ tested the improvement in model fit compared with the prior model. AIC = Akaike Information Criterion; BIC = Bayesian Information Criterion.

** *p* < .01. ***p < .001.

The results of the final model are summarized in Table 3. There were significant main effects of individuals’ political trust and countries’ levels of influence of criminal actors. In line with previous research (e.g., Letki, 2006), participants who reported higher levels of political trust compared with others in their own country also reported stronger endorsement of civic honesty. Conversely, country-level increase in the influence of criminal groups was negatively associated with individuals’ endorsement of civic honesty. The hypothesized cross-level interaction was significant, indicating that some of the heterogeneity in the slope of political trust could be accounted for by differences in the influence of criminal groups.

**Table 3:** Multilevel Model With Cross-Level Interaction

Parameters	*b*	95% CI	*SE*	*t*-ratio	*p*-value
(Intercept)	8.620	8.492, 8.748	0.064	133.56	<.001*
*Individual-level variables*					
Political Trust	0.057	0.007, 0.106	0.025	2.278	.025*
Education	0.041	0.036, 0.046	0.003	16.135	<.001*
Income	−0.003	−0.007, 0.001	0.002	−1.656	.098
Age	0.014	0.013, 0.014	0.001	48.771	<.001*
Gender	0.058	0.050, 0.067	0.004	13.449	<.001*
*Country-level indicators*					
Criminal Groups	−.166	−0.294, –0.039	0.064	−2.602	.011*
Country-level Political Trust	−0.162	−0.577, 0.253	0.204	−0.792	.431
HDI	−0.641	−3.087, 1.804	1.204	−0.533	.596
GDPpc	0.282	0.039, 0.526	0.122	2.300	.024*
Voice	−0.018	−0.284, 0.248	0.133	−0.133	.894
Stability	−0.01	−0.401, 0.201	0.152	−0.657	.513
*Cross-level interaction*					
Criminal Groups × Political Trust	−0.076	−0.112, –0.040	0.018	−4.193	<.001*
*Random effects*					
Var(country)	0.342				
Var(political trust)	0.047				
Nakagawa’s *R*^2^_marginal_/*R*^2^_conditional_	0.055/0.179				

*Note.* CI = confidence interval; *SE* = standard error; HDI = Human Development Index; GDPpc = Gross Domestic Product per capita (in US$). Gender was coded as −1 = male and 1 = female.

* Significant predictor at the specified *p*-value.

**Figure 2:**
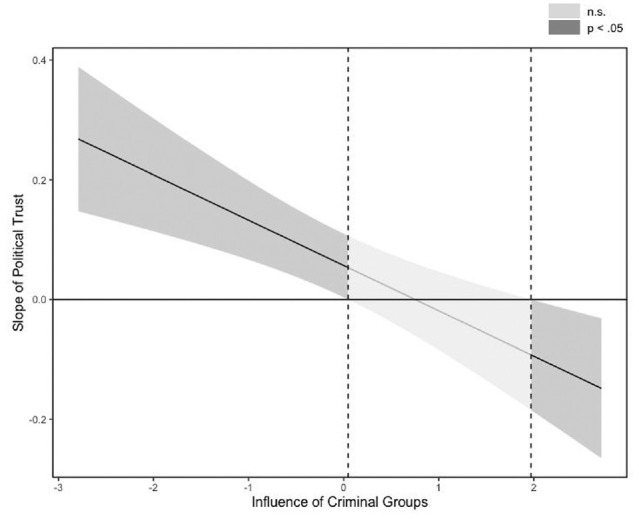
Johnson–Neyman Plot of the Focal Association Between Political Trust and Civic Honesty at the Range of Available Data of the Influence of Criminal Groups Indicator

To decompose the interaction, we employed the Johnson–Neyman (J-N) technique (Bauer & Curran, 2005; Johnson & Fay, 1950). Rather than selecting two arbitrary points, the J-N technique plots the magnitude and significance of the simple slope of the focal association (the association between individuals’ political trust and standards of civic honesty) at each level of the moderator. The plot, therefore, provides a more complete representation of the focal association across the entire range of values of the moderator. A disadvantage of the J-N technique is that it is equivalent to performing multiple comparisons, which may inflate error rates. Therefore, we adjusted the alpha level using the methodology recommended by Esarey and Sumner (2018). The J-N plot for the centered variables is shown in Figure 2.

The slope of the focal association was significant outside the 0.046 to 1.970 interval of the moderator (*SD* = .03 to *SD* = 1.44). In line with our hypothesis, the association between individuals’ political trust and civic honesty became weaker as the influence of criminal groups across countries intensified. At more extreme levels of criminal groups’ influence (i.e., more than *SD* = 1.44 from the mean), the slope was significant and negative, indicating that in those contexts individuals who reported more confidence in political and legal authorities were also more likely to justify deviations from the moral standards of civic honesty. In Figure 1, we plotted the three regions of significance calculated via the J-N against the raw values of the moderator. The white region indicates the values of the influence of criminal groups indicator in which the predicted association between political trust and civic honesty is positive and significant, whereas the light gray area indicates a weaker and nonsignificant relationship. Finally, the dark-gray area refers to the values of the moderator in which the predicted association between the variables is negative.^3^ In the Supplemental Material (Tables G–M), we further explored how the influence of different kinds of criminal groups moderated the association between individuals’ political trust and civic honesty. The cross-level interaction was replicated for all index sub-components except “Criminal Networks.” When added simultaneously, only “Foreign Actors” and “Mafias” significantly interacted with political trust, as they are distinct from the state and can effectively displace it in governance functions.

### Testing the Effects of Other Crime Rates

Criminal groups are organizations that persist over time and have a certain degree of structure. What sets these groups aside from “disorganized” criminals is their ability to influence political institutions, control territories and communities (e.g., Pinotti, 2015; Travaglino & Abrams, 2019). We conducted additional robustness tests to investigate whether differences in other crime rates moderated the association between political trust and the endorsement of civic honesty. We employed a sample of crime rate statistics published by the United Nations Office on Drugs and Crime (https://dataunodc.un.org). Although official crime rate statics are affected by limitations, such as underreporting, they provide a valuable resource for comparative research (Nivette, 2021).

We examined the effects of including a wide range of different crime rates in the model. We addressed visible and impactful offenses, including both *violent* (i.e., total rates of intentional homicide [available for 82 countries] and robbery [74 countries]) and *economic* (i.e., theft [75 countries], fraud [56 countries], burglary [69 countries], and corruption [53 countries]) offenses. Adding the main effect and cross-level interaction between each of those types of crime and individuals’ political trust did not improve the model $\chi^{2}$ (2) ≤ 4.521, *p*≥ .104. The only exceptions were the rate of intentional homicide $\chi^{2}$ (2) = 8.919, *p* = .012 and robbery $\chi^{2}$ (2) = 7.089, *p* = .029. However, in all the models, the cross-level interactions between crime rates and political trust were not significant *p*≥ .063, whereas the interaction between the indicator of influence of criminal groups and individuals’ political trust remained significant *p*≤ .034 (see the Supplemental Material).

## Discussion

The conditions that may induce individuals to endorse stronger standards of civic honesty are of substantial scientific and applied relevance. Individuals’ views of legal and political authorities—specifically, their trust and confidence in institutions—play a crucial role in driving adherence to moral and legal standards (Tyler & Jackson, 2014). Trust in authorities fosters people’s agreement with the social contract binding citizens and institutions together, and prescribing civic duties in exchange for good governance (cf. Besley, 2020). Despite the relevance of political trust in facilitating the endorsement of civic honesty, prior research has shown some heterogeneity across countries in the relationship between the two constructs (e.g., Chan et al., 2017). However, psychological research has yet to systematically address the contextual dynamics that may explain this heterogeneity.

Here, we examined an important but understudied factor affecting countries worldwide, that is the extent to which they are affected by organized criminal groups. Criminal groups are large and powerful organizations capable of exerting authority over large swathes of the population and corroding the quality and nature of institutions (Barnes, 2017; Lessing, 2020; Travaglino & Abrams, 2019). We tested the hypothesis that the association between trust and the endorsement of civic honesty would be weaker in countries more strongly influenced by such groups.

Results indicated that, in line with previous theorizing and research (e.g., Letki, 2006; Tyler, 2006), individuals’ trust in political and legal authorities and institutions was positively associated with the endorsement of civic honesty. However, our study shed light on an important variable linked to cross-country variation in this association. Specifically, the association between political trust and civic honesty significantly weakened in countries where the influence of criminal groups was stronger, in line with the idea that these groups undermined authorities’ roles as moral referents. The cross-level interaction between political trust and influence of criminal groups was robust to a series of controls involving other crime rate statistics. Although criminal groups also engage in actions such as fraud and robberies, their ability to become “organized,” gain control of territories, and their longevity distinguishes them from “disorganized” acts of crime (Pinotti, 2015).

Notably, albeit not initially predicted, we found that in countries characterized by a more extreme influence of criminal groups, the expected association between political trust and civic honesty was significant and of the opposite sign. Namely, individuals who reported more confidence and trust in authorities were also less likely to endorse standards of civic honesty and more likely to justify actions such as tax evasion, corruption, and cheating on benefits. A plausible interpretation for this finding refers to criminal groups’ capacity to “hijack” the state and subvert the nature and moral mandate of institutions. In contexts characterized by a more extreme influence of criminal groups, institutions often succumb to private and illegal interests, and public bodies may become complicit in illicit practices (Allum & Siebert, 2003; García Pinzón & Mantilla, 2021). It is, therefore, plausible that individuals who report more trust and confidence in institutions in such contexts may also be more likely to endorse immoral standards and justify illegal actions.

Trust can typically be understood as a positive expectation that others—in this case, state institutions—will act in one’s best interest (e.g., by promoting welfare among citizens). However, other facets of trust concern the predictability of others’ behavior and information certainty (Weiss et al., 2021). Therefore, in contexts with extremely high influence of criminal groups, people’s confidence in the institutions might reflect certainty about what kind of (immoral) standards to expect from the entities representing the state. Research on moral disengagement shows that individuals are more likely to rationalize unethical behavior in situations characterized by negative leadership or a general unethical climate (Hodge et al., 2013; Moore et al., 2019; Newman et al., 2020). Thus, confidence in subverted institutions might be accompanied by moral disengagement and lower civic honesty.

Criminal groups’ capacity to alter the ways citizens view state institutions may have, in turn, profound implications for democracy. In contexts where the influence of criminal groups is more extreme, lower endorsement of civic honesty may threaten the state’s ability to offer services effectively, giving rise to a negative spiral that can ultimately reinforce criminal groups’ influence. A more nuanced measurement of political trust and its facets is needed to better understand how the extreme influence of criminal groups shapes people’s understanding of confidence in institutions and, subsequently, the endorsement of civic standards.

Another important priority for future research is to use longitudinal methods and future iterations of the GI-TOC index to investigate these dynamics over time, also considering citizens’ attitudes toward illegal, criminal, and other informal practices of governance. Experimental methods should be employed to examine the articulation among the perceived influence criminal groups, individuals’ political trust and their endorsement of civic honesty (cf. Spadaro et al., 2022). Finally, as additional data quantifying the impact of criminal groups become available, researchers should assess within-countries differences in the social implications linked to these groups’ presence.

More research is also needed to identify the predictors of civic honesty in contexts where individuals’ standards are not driven by their confidence in authorities and institutions. Previous research has shown that individuals’ cultural values predict the endorsement of moral attitudes in the personal and sexual domains but not the civic one (Vauclair & Fischer, 2011). It is conceivable that cultural and personal values may acquire renewed relevance in contexts where the association between trust in authorities and civic honesty is weakened by the influence of criminal groups. Alternatively, in those contexts, individuals may be more likely to base their moral considerations on instrumental motives and cost-benefit analyses, such as the perceived likelihood of being caught or punished (Tyler, 2006).

## Conclusion

In this research, we reported evidence that the harmful influence of criminal groups in society is associated with a lower capacity of political and legal authorities to elicit positive moral standards of civic honesty among individuals. The weakened role of institutions may have dramatic consequences for the long-term viability of democracy, and is ultimately linked to lower civic cooperation and higher dishonesty. Thus, our findings reveal how criminal groups’ influence could have implications beyond economy and security and be linked to individuals’ moral attitudes in the civic context. More psychological research is needed to assess criminal groups’ wider societal impact.

Notably, as indicated by our results, expressing confidence in political and legal authorities in contexts strongly affected by criminal groups is associated with reduced standards of morality. The latter finding suggests that issues of civic honesty cannot be merely resolved by boosting people’s trust in institutions. Where institutions are fundamentally influenced by criminal groups, trust could, in fact, be associated with negative implications. This finding is consistent with the notion that, although extremely valuable, trust is not a panacea (Norris, 2022). In some contexts, critical skepticism from citizens may be warranted and beneficial.

## Supplemental Material

sj-docx-1-spp-10.1177_19485506231176615 – Supplemental material for Alternative Systems: The Interplay Between Criminal Groups’ Influence and Political Trust on Civic Honesty in the Global Context Supplemental material, sj-docx-1-spp-10.1177_19485506231176615 for Alternative Systems: The Interplay Between Criminal Groups’ Influence and Political Trust on Civic Honesty in the Global Context by Giovanni A. Travaglino, Pascal Burgmer and Alberto Mirisola in Social Psychological and Personality Science

## Notes

1. The construct considered in the present article has been labeled differently across disciplines and research areas, including “civic cooperation” (Balliet & Van Lange, 2013; Herrmann et al., 2008; Knack & Keefer, 1997), “fraud tolerance” (Knechel & Mintchik, 2022) and “law compliance” (Marien & Hooghe, 2011). We employed the label of “civic honesty” (Ariely et al., 2019; Cohn et al., 2019; Letki, 2006), reflecting our focus on individuals’ moral standards in the context of the common good.

2. In the WVS/EVS, the measure of individuals’ political orientation (from left to right) was not fielded in ten countries of the 83 available, reducing the sample size by *N* = 32,216 and removing some of the countries with the strongest influence of criminal groups (e.g., Myanmar, Iraq, and Lebanon). When included in the model, political orientation did not affect the analyses and the significance of the other variables. Therefore, we estimated a model with all 83 countries.

3. The analyses code is available at this link https://osf.io/chfx3/?view_only=feae6a948ed24f73b779c3b37342c3e9
