# Supplementary material for: Alternative Systems: The Interplay Between Criminal Groups’ Influence and Political Trust on Civic Honesty in the Global Context
Source: Soc Psychol Personal Sci. 2023 Jun 7;15(4):439–49. doi: 10.1177/19485506231176615 (PMC10984803; doi:10.1177/19485506231176615)
Supplement: sj-docx-1-spp-10.1177_19485506231176615 – Supplemental material for Alternative Systems: The Interplay Between Criminal Groups’ Influence and Political Trust on Civic Honesty in the Global Context [file sj-docx-1-spp-10.1177_19485506231176615.docx]

**APPENDIX**

Alternative Systems: The Interplay between Criminal Groups’ Influence and Political Trust on Civic Honesty in the Global Context

Giovanni A. Travaglino^1^, Pascal Burgmer^2^, Alberto Mirisola^3^

^1^ Royal Holloway, University of London

^2^ University of Southampton

^3^ University of Palermo

Figure A

*Rates of Victims of Intentional Homicide across Countries*

**
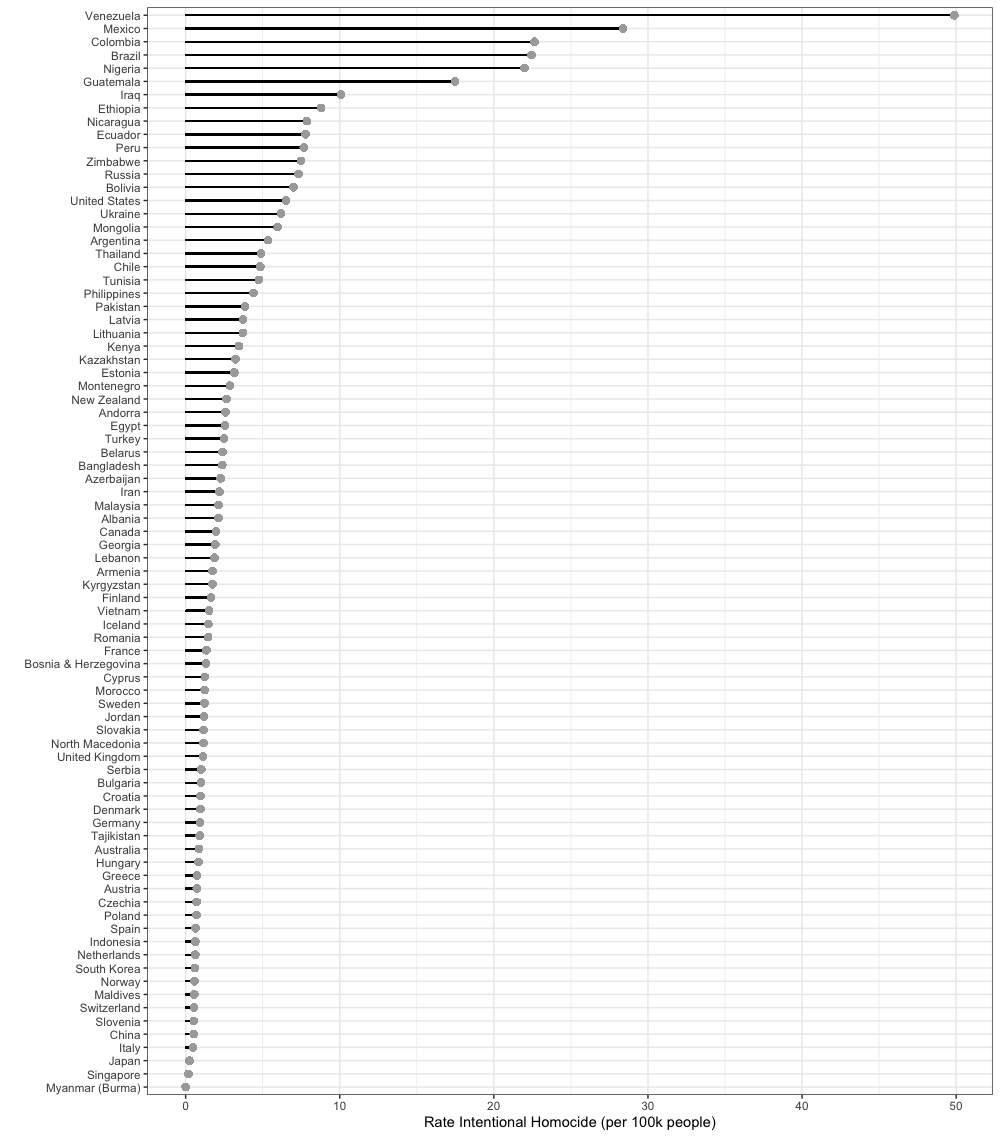
**

Note. The figure reports the rate of total intentional homicide (per 100k people) across countries.

Table A

*Multilevel Model Including the Cross-Level Interaction between Political Trust and Intentional Homicide Rates*

|  | **Moral Attitudes** | | |
| --- | --- | --- | --- |
| *Predictors* | *Estimates* | *CI* | *p* |
| Intercept | 8.72 | 8.57 – 8.88 | **<0.001** |
| Political Trust | 0.09 | 0.03 – 0.15 | **0.003** |
| Country-Level Political Trust | -0.39 | -0.83 – 0.05 | 0.083 |
| HDI | -1.39 | -3.72 – 0.95 | 0.246 |
| Gender | 0.12 | 0.10 – 0.13 | **<0.001** |
| Age | 0.01 | 0.01 – 0.01 | **<0.001** |
| Education | 0.04 | 0.04 – 0.05 | **<0.001** |
| Income | -0.00 | -0.01 – 0.00 | 0.116 |
| GDPpc | 0.36 | 0.12 – 0.61 | **0.004** |
| Voice | -0.05 | -0.30 – 0.21 | 0.722 |
| Stability | -0.08 | -0.38 – 0.23 | 0.623 |
| Rate Homocide | -0.03 | -0.05 – -0.01 | **0.013** |
| Criminal Groups | -0.13 | -0.25 – -0.00 | **0.043** |
| Political Trust*Criminal Groups | -0.06 | -0.10 – -0.02 | **0.002** |
| Political Trust*Rate Homocide | -0.01 | -0.01 – 0.00 | 0.063 |
| **Random Effects** | | | |
| σ^2^ | 2.38 | | |
| τ_00_ _country_ | 0.32 | | |
| τ_11_ _country*political trust_ | 0.04 | | |
| ρ_01_ _country_ | 0.06 | | |
| ICC | 0.12 | | |
| N _country_ | 82 | | |
| Observations | 127731 | | |
| Marginal R^2^ / Conditional R^2^ | 0.065 / 0.179 | | |

Figure B

*Rates of Victims of Robberies across Countries*

**
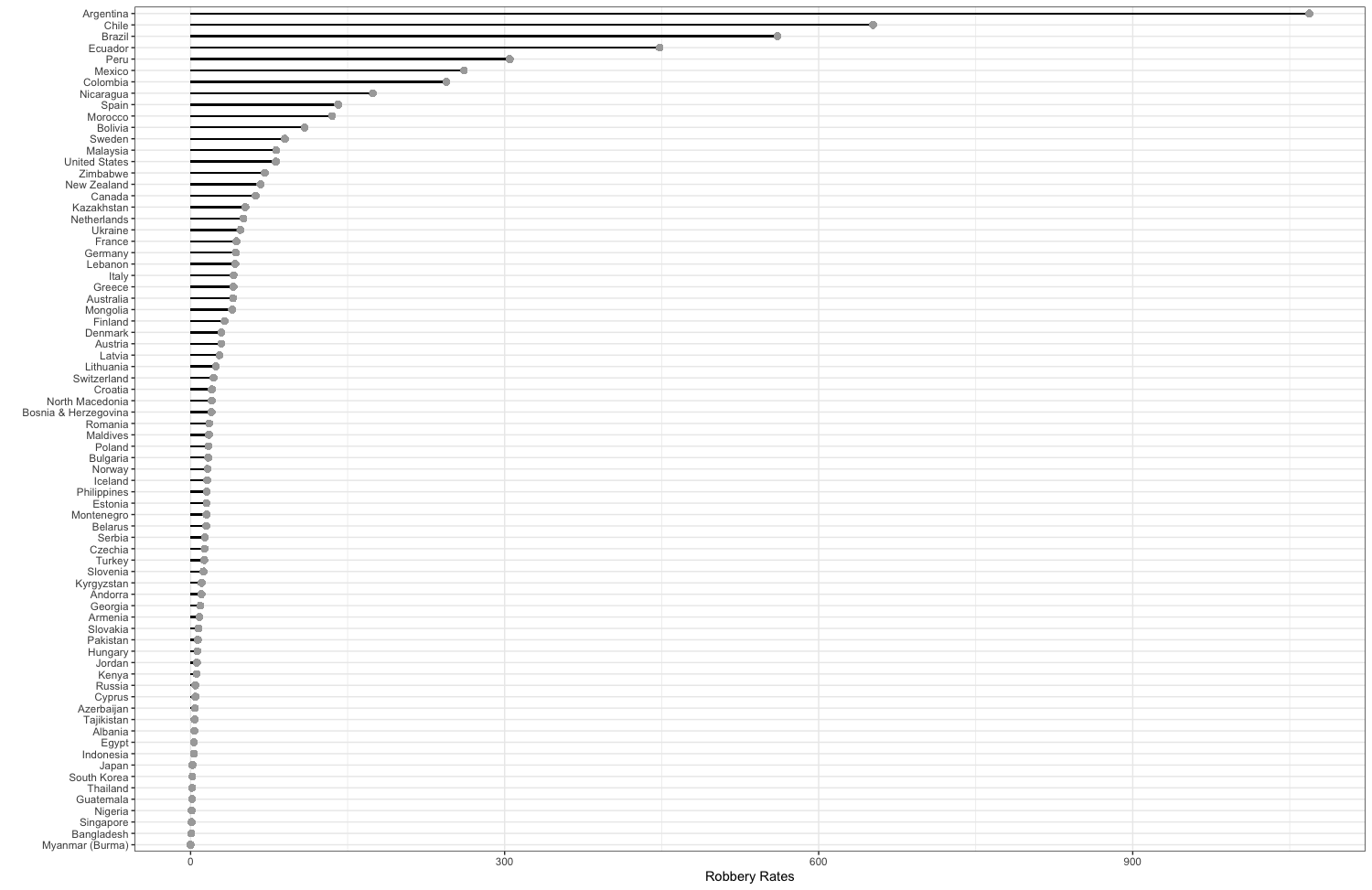
**

Note. The figure reports the rate of robberies (per 100k people) across countries.

Table B

*Multilevel Model Including the Cross-Level Interaction between Political Trust and Robbery Rates*

|  | **Moral Attitudes** | | |
| --- | --- | --- | --- |
| *Predictors* | *Estimates* | *CI* | *p* |
| Intercept | 8.60 | 8.47 – 8.73 | **<0.001** |
| Political Trust | 0.06 | 0.01 – 0.12 | **0.016** |
| Country-Level Political Trust | -0.36 | -0.82 – 0.10 | 0.128 |
| HDI | -0.73 | -3.32 – 1.86 | 0.581 |
| Gender | 0.12 | 0.11 – 0.14 | **<0.001** |
| Age | 0.01 | 0.01 – 0.01 | **<0.001** |
| Education | 0.04 | 0.03 – 0.04 | **<0.001** |
| Income | -0.00 | -0.01 – 0.00 | 0.274 |
| GDPpc | 0.23 | -0.02 – 0.49 | 0.074 |
| Voice | 0.05 | -0.24 – 0.33 | 0.754 |
| Stability | -0.04 | -0.41 – 0.32 | 0.815 |
| Robbery Rates | -0.17 | -0.29 – -0.05 | **0.007** |
| Criminal Groups | -0.13 | -0.27 – 0.00 | 0.058 |
| Political Trust* Criminal Groups | -0.07 | -0.11 – -0.03 | **<0.001** |
| Political Trust*Robbery Rates | -0.01 | -0.06 – 0.03 | 0.599 |
| **Random Effects** | | | |
| σ^2^ | 2.38 | | |
| τ_00_ _country_ | 0.32 | | |
| τ_11_ _country political trust_ | 0.05 | | |
| ρ_01_ _country_ | 0.09 | | |
| ICC | 0.12 | | |
| N _country_ | 74 | | |
| Observations | 115761 | | |
| Marginal R^2^ / Conditional R^2^ | 0.063 / 0.180 | | |

Figure C

*Rates of Theft across Countries*

**
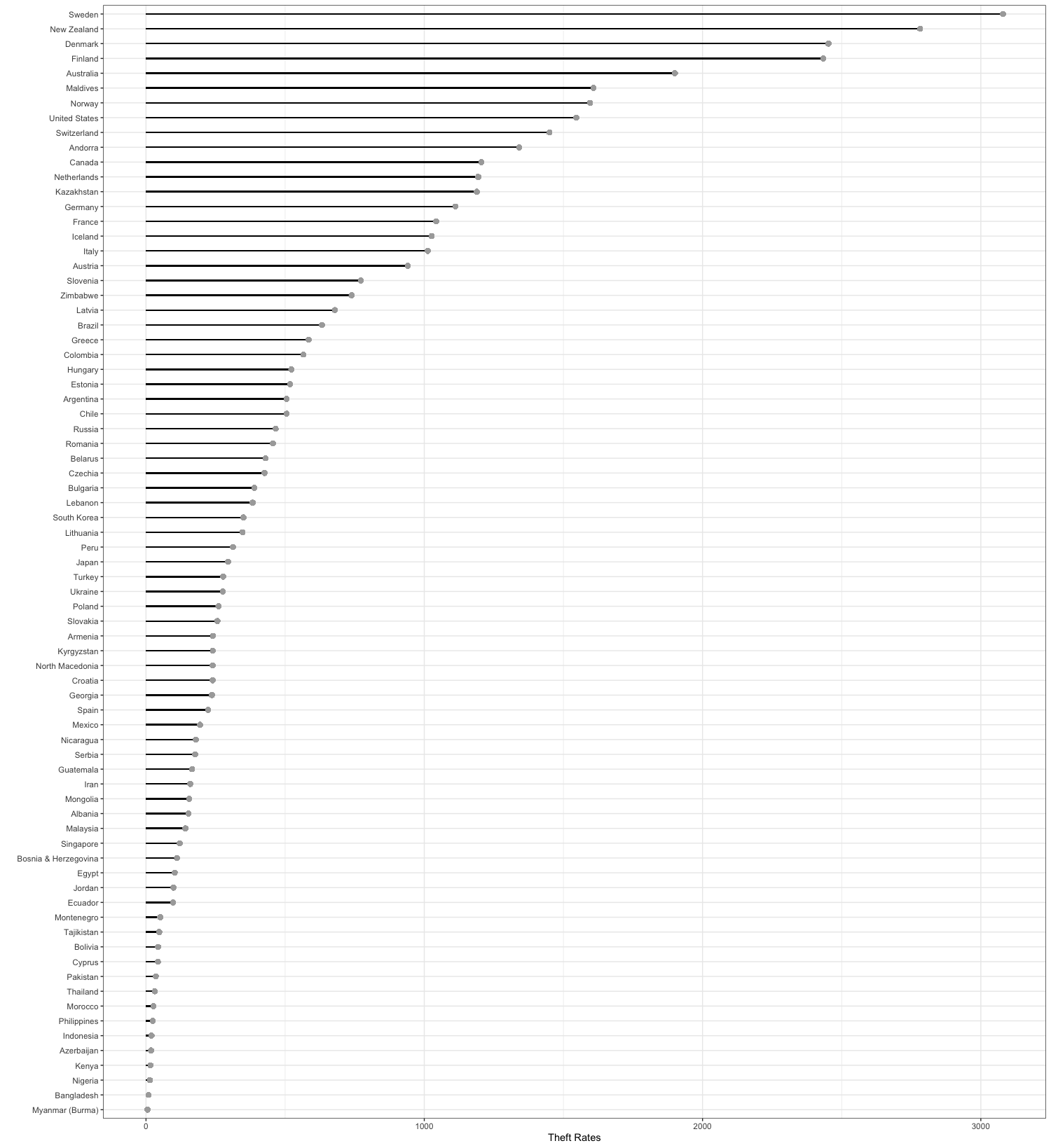
**

Note. The figure reports the rate of theft (per 100k people) across countries.

Table C

*Multilevel Model Including the Cross-Level Interaction between Political Trust and Theft Rates*

|  | **Moral Attitudes** | | |
| --- | --- | --- | --- |
| *Predictors* | *Estimates* | *CI* | *p* |
| Intercept | 8.61 | 8.47 – 8.75 | **<0.001** |
| Political Trust | 0.07 | 0.02 – 0.12 | **0.011** |
| Country-Level Political Trust | -0.21 | -0.67 – 0.26 | 0.384 |
| HDI | -0.79 | -3.36 – 1.78 | 0.546 |
| Gender | 0.12 | 0.10 – 0.14 | **<0.001** |
| Age | 0.01 | 0.01 – 0.01 | **<0.001** |
| Education | 0.04 | 0.03 – 0.05 | **<0.001** |
| Income | -0.00 | -0.01 – 0.00 | 0.210 |
| GDPpc | 0.22 | -0.07 – 0.51 | 0.133 |
| Voice | -0.06 | -0.35 – 0.23 | 0.694 |
| Stability | 0.03 | -0.34 – 0.40 | 0.875 |
| Theft Rates | 0.08 | -0.11 – 0.27 | 0.412 |
| Criminal Groups | -0.12 | -0.26 – 0.02 | 0.099 |
| Political Trust* Criminal Groups | -0.06 | -0.10 – -0.02 | **0.004** |
| Political Trust*Theft Rates | 0.02 | -0.03 – 0.08 | 0.426 |
| **Random Effects** | | | |
| σ^2^ | 2.39 | | |
| τ_00_ _country_ | 0.35 | | |
| τ_11_ _country political trust_ | 0.05 | | |
| ρ_01_ _country_ | 0.08 | | |
| ICC | 0.13 | | |
| N _country_ | 75 | | |
| Observations | 117238 | | |
| Marginal R^2^ / Conditional R^2^ | 0.055 / 0.180 | | |

Figure D

*Rates of Fraud across Countries*

**
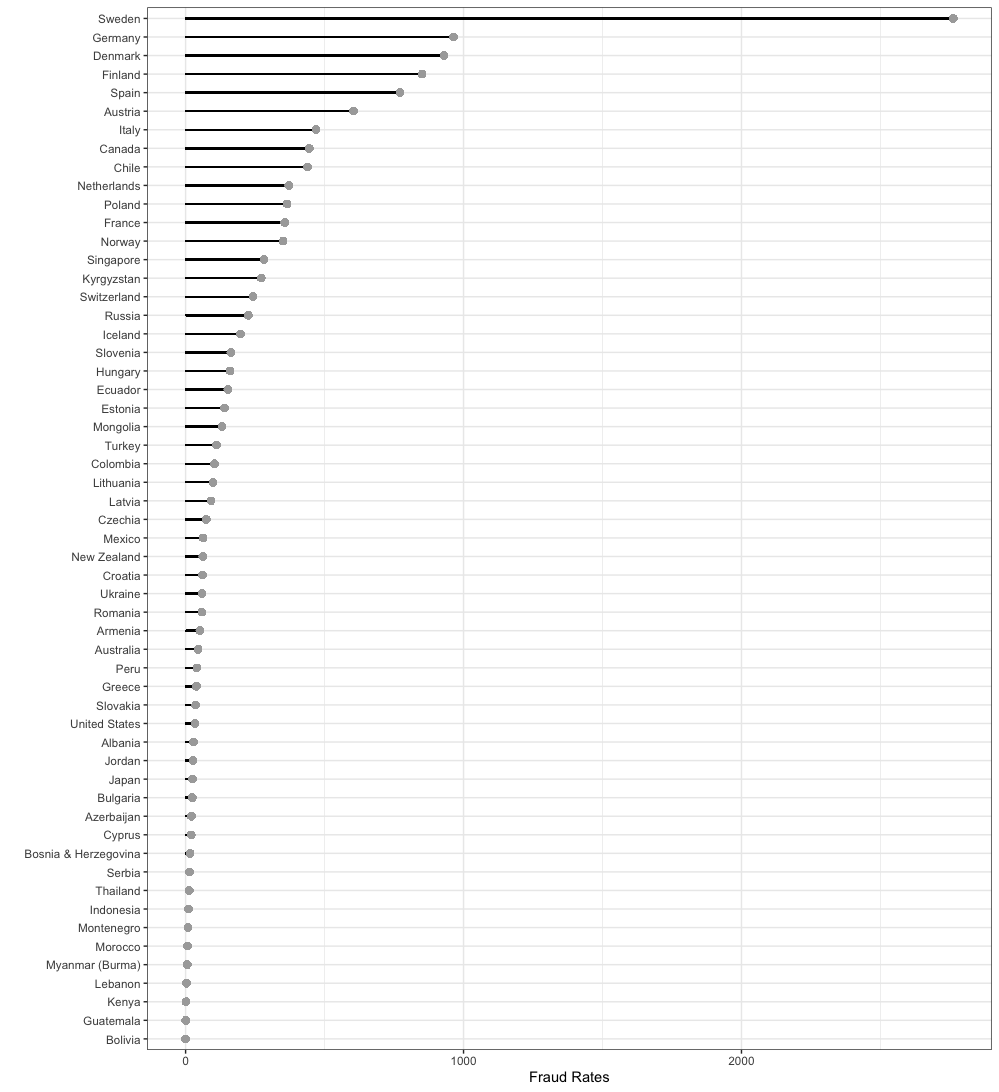
**

Note. The figure reports the rate of fraud (per 100k people) across countries.

Table D

*Multilevel Model Including the Cross-Level Interaction between Political Trust and Fraud Rates*

|  | **Moral Attitudes** | | |
| --- | --- | --- | --- |
| *Predictors* | *Estimates* | *CI* | *p* |
| Intercept | 8.55 | 8.41 – 8.70 | **<0.001** |
| Political Trust | 0.07 | 0.01 – 0.13 | **0.027** |
| Country-Level Political Trust | 0.43 | -0.09 – 0.96 | 0.106 |
| HDI | 4.34 | 1.15 – 7.53 | **0.008** |
| Gender | 0.14 | 0.12 – 0.16 | **<0.001** |
| Age | 0.02 | 0.01 – 0.02 | **<0.001** |
| Education | 0.04 | 0.03 – 0.04 | **<0.001** |
| Income | 0.00 | -0.00 – 0.01 | 0.340 |
| GDPpc | -0.07 | -0.33 – 0.18 | 0.575 |
| Voice | -0.11 | -0.44 – 0.23 | 0.529 |
| Stability | 0.13 | -0.25 – 0.51 | 0.510 |
| Fraud Rates | -0.05 | -0.18 – 0.09 | 0.517 |
| Criminal Groups | 0.02 | -0.12 – 0.16 | 0.770 |
| Political Trust* Criminal Groups | -0.07 | -0.12 – -0.03 | **0.001** |
| Political Trust*Fraud Rates | 0.01 | -0.04 – 0.07 | 0.617 |
| **Random Effects** | | | |
| σ^2^ | 2.32 | | |
| τ_00_ _country_ | 0.23 | | |
| τ_11_ _country political trust_ | 0.05 | | |
| ρ_01_ _country_ | -0.11 | | |
| ICC | 0.10 | | |
| N _country_ | 56 | | |
| Observations | 93291 | | |
| Marginal R^2^ / Conditional R^2^ | 0.077 / 0.167 | | |

Figure E

*Rates of Burglaries across Countries*


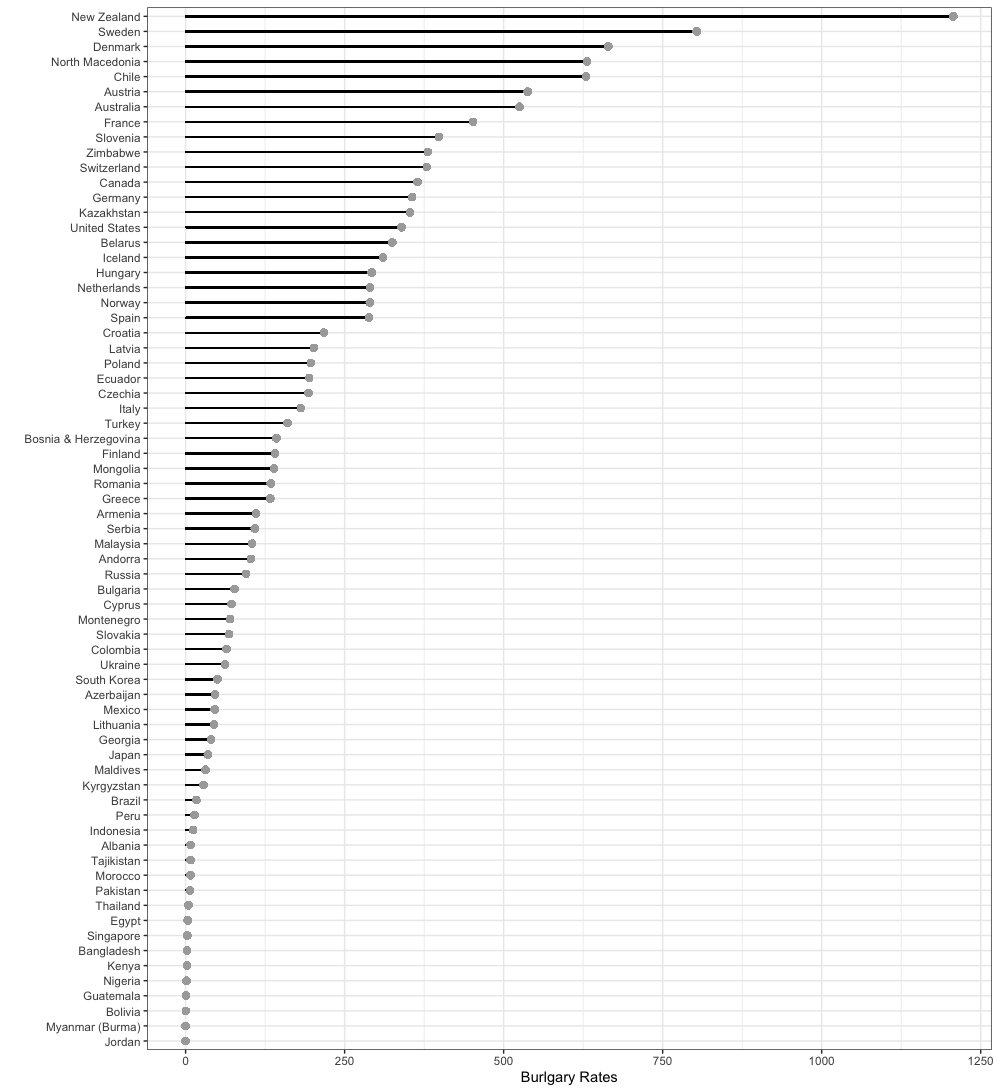


Note. The figure reports the rate of burglaries (per 100k people) across countries.

Table E

*Multilevel Model Including the Cross-Level Interaction between Political Trust and Burglaries Rates*

|  | **Moral Attitudes** | | | |
| --- | --- | --- | --- | --- |
| *Predictors* | *Estimates* | *CI* | | *p* |
| Intercept | 8.64 | 8.50 – 8.78 | | **<0.001** |
| Political Trust | 0.08 | 0.03 – 0.13 | | **0.002** |
| Country-Level Political Trust | 0.01 | -0.49 – 0.51 | | 0.967 |
| HDI | -0.95 | -3.63 – 1.73 | | 0.487 |
| Gender | 0.12 | 0.10 – 0.14 | | **<0.001** |
| Age | 0.01 | 0.01 – 0.01 | | **<0.001** |
| Education | 0.04 | 0.03 – 0.04 | | **<0.001** |
| Income | 0.00 | -0.00 – 0.00 | | 0.892 |
| GDPpc | 0.20 | -0.07 – 0.47 | | 0.139 |
| Voice | 0.04 | -0.27 – 0.35 | | 0.810 |
| Stability | 0.02 | -0.36 – 0.40 | | 0.921 |
| Burlgary Rates | -0.00 | -0.15 – 0.15 | | 0.964 |
| Criminal Groups | -0.09 | -0.23 – 0.06 | | 0.231 |
| Political Trust*Criminal Groups | -0.04 | -0.09 – -0.00 | | **0.036** |
| Political Trust*Burglary Rates | 0.04 | -0.01 – 0.09 | | 0.145 |
| **Random Effects** | | | | |
| σ^2^ | 2.34 | | | |
| τ_00_ _country_ | 0.32 | | | |
| τ_11_ _country political trust_ | 0.04 | | | |
| ρ_01_ _country_ | 0.02 | | | |
| ICC | 0.13 | | | |
| N _country_ | 69 | | | |
| Observations | 110045 | | | |
| Marginal R^2^ / Conditional R^2^ | 0.047 / 0.167 | |  |  |

Figure F

*Rates of Corruption across Countries*


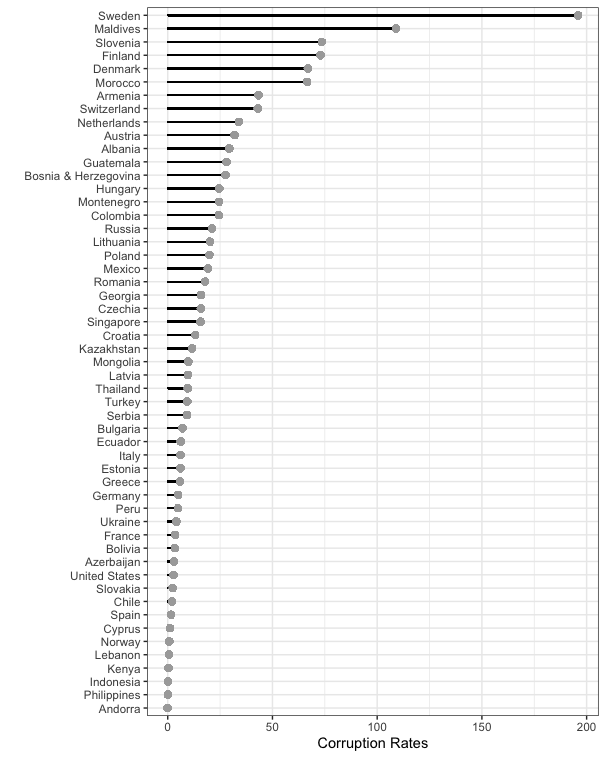


Note. The figure reports the rate of corruption (per 100k people) across countries.

Table F

|  | **Moral Attitudes** | | |
| --- | --- | --- | --- |
| *Predictors* | *Estimates* | *CI* | *p* |
| Intercept | 8.42 | 8.25 – 8.59 | **<0.001** |
| Political Trust | 0.07 | 0.00 – 0.13 | **0.048** |
| Country-Level Political Trust | -0.25 | -0.78 – 0.28 | 0.349 |
| HDI | 5.45 | 1.97 – 8.92 | **0.002** |
| Gender | 0.12 | 0.10 – 0.14 | **<0.001** |
| Age | 0.01 | 0.01 – 0.01 | **<0.001** |
| Education | 0.03 | 0.03 – 0.04 | **<0.001** |
| Income | 0.01 | 0.00 – 0.01 | **0.038** |
| GDP | -0.02 | -0.31 – 0.27 | 0.880 |
| Voice | -0.20 | -0.54 – 0.14 | 0.248 |
| Stability | 0.17 | -0.22 – 0.56 | 0.395 |
| Corruption Rates | 0.09 | -0.04 – 0.23 | 0.175 |
| Influence of Criminal Groups | -0.04 | -0.18 – 0.10 | 0.582 |
| Political Trust*Influence of Criminal Groups | -0.08 | -0.12 – -0.03 | **0.002** |
| Political Trust*Corruption Rates | -0.00 | -0.07 – 0.06 | 0.905 |
| **Random Effects** | | | |
| σ^2^ | 2.35 | | |
| τ_00_ _country_ | 0.29 | | |
| τ_11_ _country.political_trust_ | 0.05 | | |
| ρ_01_ _country_ | -0.16 | | |
| ICC | 0.12 | | |
| N _country_ | 53 | | |
| Observations | 86840 | | |
| Marginal R^2^ / Conditional R^2^ | 0.094 / 0.200 | | |

*Multilevel Model Including the Cross-Level Interaction between Political Trust and Corruption Rates*

**Analyses of GI-TOC Index Subcomponents**

To examine the moderating effects of different types of organized criminal groups, we reanalysed the data employing each of the four subcomponents of the “Influence of Criminal Groups” GI-TOC indicator, namely *Mafia-style Actors,* *State-embedded* *Actors*, *Criminal Networks* and *Foreign Actors* (see the main article for a description; cf. also Methodology, n.d.). Moreover, we modelled the effects of all four subcomponents simultaneously. Results from these models are reported in Tables G-M below.

The cross-level interaction described in the main article was replicated with each subcomponent except for the Criminal Networks one. Criminal Networks are characterised by lower territorial control than other organized criminal groups included in the GI-TOC indicator. Thus, these groups are less likely to displace the state and exert governance over communities. Interestingly, in the model including all sub-components, only the interactions between political trust and the *Mafia-style Actors* and *Foreign Actors* subcomponents remained significant. This latter finding suggests that the effect of the cross-level interaction between Criminal Groups’ influence and political trust may be mainly driven by the influence in a territory of alternative and illegal systems of power that are simultaneously distinct from the state and can be recognised by people (owing to features such as a clear structure, a hierarchy and a known name).

Table G

*Model with Mafia-style Actors*

|  | **Moral Attitudes** | | |
| --- | --- | --- | --- |
| *Predictors* | *Estimates* | *CI* | *p* |
| Intercept | 8.62 | 8.49 – 8.75 | <0.001 |
| Political Trust | 0.06 | 0.01 – 0.11 | 0.027 |
| Country-Level Political Trust | -0.12 | -0.52 – 0.29 | 0.571 |
| HDI | -0.68 | -3.07 – 1.72 | 0.581 |
| Gender | 0.06 | 0.05 – 0.07 | <0.001 |
| Age | 0.01 | 0.01 – 0.01 | <0.001 |
| Education | 0.04 | 0.04 – 0.05 | <0.001 |
| Income | -0.00 | -0.01 – 0.00 | 0.103 |
| GDP | 0.31 | 0.07 – 0.56 | 0.012 |
| Voice | 0.01 | -0.25 – 0.28 | 0.936 |
| Stability | -0.05 | -0.35 – 0.24 | 0.733 |
| Mafia-style Actors | -0.07 | -0.14 – -0.00 | 0.041 |
| Political Trust*Mafia-style Actors | -0.05 | -0.07 – -0.02 | <0.001 |
| Random Effects | | | |
| σ^2^ | 2.37 | | |
| τ_00_ _country_ | 0.35 | | |
| τ_11_ _country.political_trust_ | 0.05 | | |
| ρ_01_ _country_ | 0.06 | | |
| ICC | 0.13 | | |
| N _country_ | 83 | | |
| Observations | 128839 | | |
| Marginal R^2^ / Conditional R^2^ | 0.049 / 0.177 | | |

Table H

*Model with State-embedded Actors*

|  | **Moral Attitudes** | | |
| --- | --- | --- | --- |
| *Predictors* | *Estimates* | *CI* | *p* |
| Intercept | 8.62 | 8.49 – 8.75 | <0.001 |
| Political Trust | 0.06 | 0.01 – 0.11 | 0.030 |
| Country-Level Political Trust | -0.21 | -0.62 – 0.20 | 0.308 |
| HDI | -0.57 | -3.02 – 1.87 | 0.646 |
| Gender | 0.06 | 0.05 – 0.07 | <0.001 |
| Age | 0.01 | 0.01 – 0.01 | <0.001 |
| Education | 0.04 | 0.04 – 0.05 | <0.001 |
| Income | -0.00 | -0.01 – 0.00 | 0.095 |
| GDP | 0.21 | -0.05 – 0.47 | 0.111 |
| Voice | -0.09 | -0.38 – 0.19 | 0.523 |
| Stability | -0.02 | -0.31 – 0.26 | 0.866 |
| State-embedded Actors | -0.10 | -0.20 – -0.00 | 0.045 |
| Political Trust*State-embedded Actors | -0.03 | -0.05 – -0.01 | 0.003 |
| Random Effects | | | |
| σ^2^ | 2.37 | | |
| τ_00_ _country_ | 0.36 | | |
| τ_11_ _country.political_trust_ | 0.05 | | |
| ρ_01_ _country_ | 0.14 | | |
| ICC | 0.14 | | |
| N _country_ | 83 | | |
| Observations | 128839 | | |
| Marginal R^2^ / Conditional R^2^ | 0.051 / 0.180 |  |  |

Table I

*Model with Criminal Networks*

|  | **Moral Attitudes** | | |
| --- | --- | --- | --- |
| *Predictors* | *Estimates* | *CI* | *p* |
| Intercept | 8.62 | 8.49 – 8.75 | <0.001 |
| Political Trust | 0.06 | 0.00 – 0.11 | 0.036 |
| Country-Level Political Trust | -0.15 | -0.56 – 0.26 | 0.472 |
| HDI | -1.14 | -3.56 – 1.27 | 0.354 |
| Gender | 0.06 | 0.05 – 0.07 | <0.001 |
| Age | 0.01 | 0.01 – 0.01 | <0.001 |
| Education | 0.04 | 0.04 – 0.05 | <0.001 |
| Income | -0.00 | -0.01 – 0.00 | 0.099 |
| GDP | 0.29 | 0.04 – 0.54 | 0.021 |
| Voice | 0.01 | -0.26 – 0.28 | 0.929 |
| Stability | 0.04 | -0.26 – 0.33 | 0.802 |
| Criminal Networks | -0.02 | -0.13 – 0.08 | 0.675 |
| Political Trust*Criminal Networks | -0.03 | -0.07 – 0.00 | 0.055 |
| **Random Effects** | | | |
| σ^2^ | 2.37 | | |
| τ_00_ _country_ | 0.37 | | |
| τ_11_ _country.political_trust_ | 0.05 | | |
| ρ_01_ _country_ | 0.14 | | |
| ICC | 0.14 | | |
| N _country_ | 83 | | |
| Observations | 128839 | | |
| Marginal R^2^ / Conditional R^2^ | 0.042 / 0.177 | | |

Table L

*Model with Foreign Actors*

|  | Moral Attitudes | | |
| --- | --- | --- | --- |
| *Predictors* | *Estimates* | *CI* | *p* |
| Intercept | 8.62 | 8.49 – 8.75 | <0.001 |
| Political Trust | 0.06 | 0.00 – 0.11 | 0.032 |
| Country-Level Political Trust | -0.14 | -0.53 – 0.26 | 0.499 |
| HDI | -1.16 | -3.50 – 1.17 | 0.329 |
| Gender | 0.06 | 0.05 – 0.07 | <0.001 |
| Age | 0.01 | 0.01 – 0.01 | <0.001 |
| Education | 0.04 | 0.04 – 0.05 | <0.001 |
| Income | -0.00 | -0.01 – 0.00 | 0.099 |
| GDP | 0.34 | 0.09 – 0.58 | 0.006 |
| Voice | 0.03 | -0.23 – 0.29 | 0.804 |
| Stability | -0.01 | -0.29 – 0.26 | 0.921 |
| Foreign Actors | -0.12 | -0.21 – -0.03 | 0.006 |
| Political Trust*Foreign Actors | -0.05 | -0.09 – -0.02 | 0.003 |
| Random Effects | | | |
| σ^2^ | 2.37 | | |
| τ_00_ _country_ | 0.34 | | |
| τ_11_ _country.political_trust_ | 0.05 | | |
| ρ_01_ _country_ | 0.06 | | |
| ICC | 0.13 | | |
| N _country_ | 83 | | |
| Observations | 128839 | | |
| Marginal R^2^ / Conditional R^2^ | 0.052 / 0.176 | | |

Table M

*Model with all Subcomponents*

|  | **Moral Attitudes** | | |
| --- | --- | --- | --- |
| Predictors | Estimates | CI | p |
| Intercept | 8.62 | 8.50 – 8.74 | <0.001 |
| Political Trust | 0.06 | 0.01 – 0.10 | 0.020 |
| Country-Level Political Trust | -0.13 | -0.52 – 0.27 | 0.527 |
| HDI | -0.35 | -2.73 – 2.03 | 0.773 |
| Gender | 0.06 | 0.05 – 0.07 | <0.001 |
| Age | 0.01 | 0.01 – 0.01 | <0.001 |
| Education | 0.04 | 0.04 – 0.05 | <0.001 |
| Income | -0.00 | -0.01 – 0.00 | 0.099 |
| GDP | 0.31 | 0.05 – 0.57 | 0.019 |
| Voice | -0.04 | -0.33 – 0.25 | 0.776 |
| Stability | -0.08 | -0.37 – 0.21 | 0.591 |
| Foreign Actors | -0.11 | -0.19 – -0.02 | 0.019 |
| State-embedded Actors | -0.05 | -0.16 – 0.06 | 0.360 |
| Criminal Networks | 0.07 | -0.04 – 0.18 | 0.192 |
| Mafia Actors | -0.06 | -0.14 – 0.01 | 0.096 |
| Political Trust*Foreign Actors | -0.04 | -0.07 – -0.00 | 0.025 |
| Political Trust*State-embedded Actors | -0.02 | -0.05 – 0.00 | 0.080 |
| Political Trust*Criminal Networks | 0.02 | -0.02 – 0.06 | 0.334 |
| Political Trust*Mafia-style Actors | -0.04 | -0.06 – -0.01 | 0.009 |
| **Random Effects** | | | |
| σ^2^ | 2.37 | | |
| τ_00_ _country_ | 0.32 | | |
| τ_11_ _country.political_trust_ | 0.04 | | |
| ρ_01_ _country_ | -0.03 | | |
| ICC | 0.12 | | |
| N _country_ | 83 | | |
| Observations | 128839 | | |
| Marginal R^2^ / Conditional R^2^ | 0.061 / 0.178 | | |
